# Content related with preschool children's health on Facebook: a study of parental informational needs

**DOI:** 10.1093/eurpub/ckac129.572

**Published:** 2022-10-25

**Authors:** I Liepinaite, D Austys

**Affiliations:** Department of Public Health, Institute of Health Sciences, Faculty of Medicine, Vilnius University, Vilnius, Lithuania

## Abstract

**Background:**

Little is known how Lithuanian mothers use social networks to seek information about preschool children's health. This study aimed to identify health topics of the posts, characteristics of the most engaging posts, and practices shared in the comments.

**Methods:**

This study included all accessible posts published in 2021 on one Facebook group with more than 21 000 Lithuanian mothers. In total, 1674 posts and 3192 comments on the most engaged group of posts (gastrointestinal disorders) were analysed. Number and type of reactions to posts, categories of health topics, aim, form, tone, and structure of the posts, also practice type on comment were registered.

**Results:**

Among all posts, 72.9% were related to children's health. The most common health topics included injuries (17.1%), healthcare (15.1%), gastrointestinal disorders (9.7%), allergies and skin diseases (9.7%), nutrition, physical activity and health promotion (9.3%), nurture (8.9%). The most common form was text only (48.7%), repetitive aim was to ask about personal experience (47.5%), and recommendations (35.5%). Larger numbers of reactions achieved posts with sensitive tone, including appeal, child's health description, gratitude in advance, and written in Lithuanian (p < 0.05). Comments with healthy lifestyle recommendations, recommendations for medication, also recommendations to treat children by themselves, to visit physicians accounted for 73.6%, 26.4%, 36.0% and 10.8% respectively.

**Conclusions:**

Main topics of children's health on Facebook for Lithuanian mothers are injuries, healthcare, gastrointestinal disorders, allergies, skin diseases, nutrition, physical activity, health promotion, nurture. Posts with sensitive tone, appeal, situation description, gratitude, written in Lithuanian achieve the largest number of reactions. Comments most frequently include advices to treat children at home, also healthy lifestyle recommendations.

**Key messages:**

• Lithuanian mothers most frequently seek advices regarding preschool children’s injuries, healthcare, gastrointestinal issues, allergies and skin diseases, and nurture.

• Publication of posts including text only, sensitive tone, greetings, situation description, and gratitude might help to promote health more effectively.

